# Enabling near real time use of wildlife necropsy data: Text-mining approaches to derive interactive dashboard displays

**DOI:** 10.1371/journal.pone.0331210

**Published:** 2025-09-19

**Authors:** Stefan Saverimuttu, Kate McInnes, Kristin Warren, Lian Yeap, Stuart Hunter, Brett Gartrell, An Pas, James Chatterton, Bethany Jackson

**Affiliations:** 1 New Zealand Center for Conservation Medicine, Auckland Zoo, Auckland, New Zealand; 2 Centre for Biosecurity and One Health, Harry Butler Institute, Murdoch University, Perth, Australia; 3 Department of Conservation/Te Papa Atawhai, Nelson, New Zealand; 4 Centre for Terrestrial Ecosystem Science and Sustainability, Harry Butler Institute, Murdoch University, Murdoch, Australia; 5 Institute of Veterinary, Animal and Biomedical Sciences, Massey University, Wellington, New Zealand; University of Nottingham, UNITED KINGDOM OF GREAT BRITAIN AND NORTHERN IRELAND

## Abstract

Manual review of necropsy records through close reading and collation is a time-consuming process, leading to delays in knowledge acquisition, communication of findings, and subsequent actions. Text-mining techniques offer a means to reduce these barriers by automating the extraction of information from large volumes of free-text clinical reports, minimizing the need for manual review. Additionally, interactive dashboards enable end users to interrogate data dynamically, tailoring analyses to their specific needs and objectives. Here, we describe the principles underlying an application designed to extract and visualize information from free-text necropsy records within the Wildbase Pathology register. Reflecting the structure of a traditional necropsy review—where each record is examined in detail to identify and collate key observations—the application is divided into three sections. The first allows a user to upload a dataset in comma separated value format as downloaded from the Wildbase Pathology Register. A user can then filter and interrogate selected signalment variables of the population within this dataset. The second section uses established text-mining calculations of word correlations and Latent Dirichlet Allocation to generate visualisations to give a user a subjective sense of common themes found within the uploaded data. The third and final section uses a custom rule-based algorithm to identify and quantify positive occurrences of clinicopathologic findings as input by an end user. The foundational methods employed in this application have the potential for broader application in veterinary and medical pathology, facilitating more efficient and timely access to critical insights.

## Introduction

Statistical reviews of wildlife morbidity and mortality data can derive useful insights into important wildlife health trends [1–4], which in turn can refine and focus species management and research. However, unlike production animal health data, which are generally systematically collected and more statistically robust in review [5,6], many wildlife morbidity and mortality datasets are opportunistically obtained with implicit issues relating to selection bias and external validity. Thus, there may be stark differences between the sampled and free-living populations [7]. Further, standardisation in reporting of wildlife health data is often lacking across studies, creating challenges in data comparison and aggregation [8]. In production animals, regular reviews of systematically collected health data are used to identify changing disease patterns which have implications for productivity and animal welfare, as well as economic consequences such as access to export markets [9]. In contrast, the goals of wildlife health monitoring through necropsy databases appear to centre around informing conservation policy and identifying emerging infectious disease which may pose threats to wildlife, domestic animal, or human health at large [10,11]. Irrespective of the goals, data collection without analysis severely limits the value of collection efforts.

Barriers to the routine analysis of wildlife necropsy datasets span time and labour costs, and the complications of extrapolating data to the broader population of interest. The labour-intensive manual approach used in wildlife necropsy reviews mirrors the well-recognised time cost of reviewing free-text clinical records in human medicine [12]. Structural issues of datasets, ranging from biases and confounding to missing and redundant data [10,13–15], further increase the temporal, fiscal, and personnel demands of conducting a review. Opportunistic, passively acquired necropsy databases often lack external validity due to selection/admission bias [1,4], with submissions influenced by visibility and location of a species, alongside research and conservation priorities that impact access to specific species or areas. The result is that species of low threat status, remote geographic range, and/or a cryptic lifestyle may have poorer representation within such a database [15]. Careful analysis of the data may reveal such skews in species demographics. However, without an understanding of variances in survey efforts and motivations, their causes may remain undetermined [3]. Beyond the impact of bias, issues with record completeness and consistency of reporting are regularly encountered [2,16,17]. At best, such issues may lead to a reduced set of validated data for use. At worst, they will result in limitations to conclusions drawn from the dataset. Together, these issues limit reviews to sporadic, retrospective snapshots of the represented populations*.* The value of necropsy reviews to wildlife management would be improved if their data were able to be analysed and presented on a regular, systematic basis as often appears to be the case in an agricultural context [5,6].

Effective use of similar data in other industries demonstrates that the obstacles surrounding timely and regular review of wildlife necropsy data can be overcome [12,18–20]. Through embracing modern approaches to free-text data analysis and display, there is potential to leverage the spectrum of unstructured to structured data to inform wildlife conservation in a more contemporaneous and less labour-intensive manner. Text-mining is the use of common data wrangling and visualisation methodologies to gain insights from natural language texts rather than traditional numeric or tabular data [21]. These techniques are increasingly being used in human medical research for the rapid analysis of free-text clinical records [12], and examples of their use already exist within the veterinary literature [22–25]. Knowledge discovery through text-mining [26] may alleviate much of the time cost of performing a traditional review of wildlife health data. However, to complement data analysis, we need user-friendly data visualisation techniques. The utility of real-time, dashboard style reporting of data has entered the public zeitgeist with its widespread use in relation to the SARS CoV-2 pandemic [27]. The application of dashboards to visualise data analyses of wildlife necropsy databases can reduce the need for professionals in wildlife health and management to be proficient in underlying text-mining techniques. Together, text-mining and dashboard style reporting not only have the potential to improve access to trends in wildlife necropsy data but may also highlight areas where the acquisition and storage of these data may be improved.

**Fig 1:**
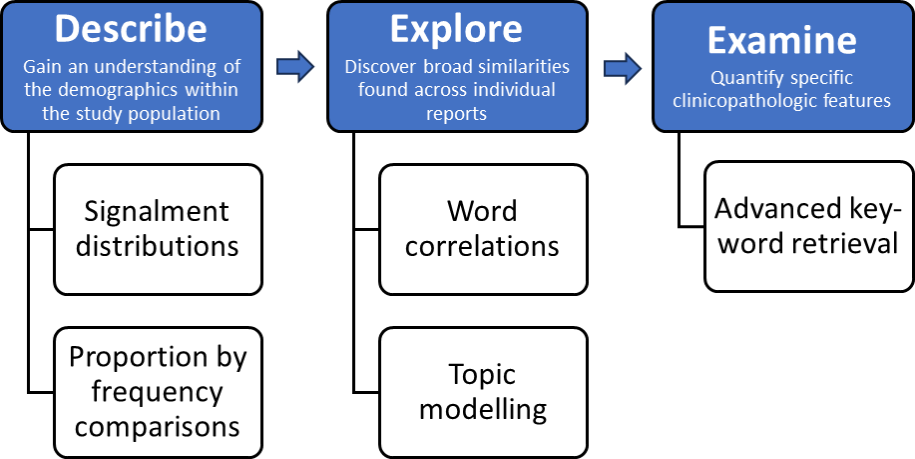
Intended workflow of the application, DEE, for extraction and display of information from free-text wildlife necropsy data. A three-step process to facilitate rapid interrogation of large numbers of necropsy reports downloaded from the Wildbase Pathology Register of New Zealand.

The Wildbase Pathology Register contains necropsy reports of wildlife from around New Zealand, making it an immense resource of opportunistically collected wildlife health data for the region. The necropsy reports themselves are organised in a standardised form separating sections such as signalment characteristics, history, gross and histopathologic findings, alongside ancillary diagnostics. However, data input into each of these sections is largely free-text in nature. These characteristics mean the utility of its contents is reduced by the constraints of needing a traditional, manual necropsy review process to determine data trends. This paper describes the creation of an application utilising text-mining and dashboard display to access knowledge within large swaths of necropsy records downloaded from the Wildbase Pathology Register. Titled ‘DEE’, an acronym for ‘*Describe*, *Explore*, *Examine*’, steps within the application intend to mimic the general workflow observed in at least a proportion of published necropsy reviews (Fig 1) without manual reading or compilation [17,28,29]. Results are then presented in an interactive dashboard style display for ease of use. The primary aim of the application was to enable end-users (professionals in wildlife health or related fields) to describe common trends which are more likely to influence wildlife management and future research. A secondary aim was to use the data displayed in the dashboard to identify issues in data input that could be resolved to enhance future analyses. For example, redundancy of terms or data fields that could be restricted or validated.

## Implementation

### Application target data source

The Massey University Veterinary School, in conjunction with Wildbase Pathology, curate and maintain the online database of native wildlife necropsy reports which are available in the Wildbase Pathology Register. Through an agreement with the New Zealand Department of Conservation, native wildlife specimens are submitted to Wildbase Pathology from around the country, and their associated records are stored in this database. Specimens are generally submitted whole to Wildbase Pathology for necropsy; however, collaborating institutions may perform the gross necropsy and submit only formalin fixed tissue samples alongside a report of the gross examination. Individual necropsy reports are entered into a standard online form hosted within the Wildbase Pathology Register itself. The form is semi-structured with drop down lists provided for entering specific signalment (e.g., species, age classification, sex etc), location and time series data alongside some capacity to add values to these lists if required. Despite the intent to standardise entry of these data, historical modifications to these lists have resulted in a multitude of redundancies. Larger free-text spaces are provided for entering findings of the various stages of necropsy investigation (e.g., gross pathology, histopathology, ancillary diagnostic test results, differential and final diagnoses). It is important to note that all such fields are optional, allowing for variability between pathologists in reporting style, as well as typographical errors or synonymous terms to be used. Stored records can be accessed using some native search capability.

Individual records are identified with a unique accession number assigned on entry, providing one method of retrospective access; however, it requires prior knowledge of this accession number. Alternatively, the entire database may be filtered by taxonomic classification, time-period or via a simple keyword search. While this does provide some exploratory functionality, the free-text nature of the records complicates the usefulness of these features. For example, searching for ‘pneumonia’ will result in records stating that evidence for pneumonia was found alongside those explicitly stating it was not. This makes the simple keyword search functionality useful for finding individual or small groups of records, yet it provides minimal ability to examine patterns of submission and pathology within large collections as with a large-scale necropsy review. The Wildbase Pathology Register does allow export of collections of records to comma separated value (.csv) format which may assist in examination of such a collection following manual review of the dataset.

### Development of software to interrogate necropsy data

The application for interrogation of the Wildbase Pathology Register’s necropsy data was built in the *Shiny* [30] package created by *R-studio* [31] for the *R* software environment (version 4.1.0 ‘Camp Pontanezen’) [32]. The application can be run in browser at the following URL: https://stefansav.shinyapps.io/necropsy-text-mining-2/. For posterity and transparency, source files for the application can be accessed via the GitHub repository at the following URL: https://github.com/SavStefan/Enabling-near-real-time-use-of-wildlife-necropsy-data or in the supporting information (S1-S3 File). An anonymised sample datafile is also included at this repository and in the supporting information (S1 Dataset). Pathological descriptions within this sample datafile are drawn from a random selection of real records within the Wildbase Pathology Register. As such, they exemplify real-world necropsy records; however, any trends observed are coincidental and not reflective of the Wildbase Pathology Register as a whole. Further, this datafile has been used to generate all figures which are presented here as sample outputs from the application. Source files licenced under GNU General Public Licence Version 3.

### Aesthetic theming

Navigation through the application’s functions is facilitated by a navigation bar with nested tabs for separate functions. The navigation bar separates the application into three discrete and sequential sections inspired by the general process of a manual necropsy review (Fig 1). Interactive features of each page are provided to the user via a sidebar. Standard aesthetic theming as default within the *Shiny* package was largely maintained, with judicious use of html script to allow inclusion of basic user instructions and spacing of visualisations to enhance readability.

### Script structure

The code scripts for the application were organised into three discrete ‘.R’ files. A ‘global.R’ file containing all the packages was required to run the functionality of the application. A ‘ui.R’ file contained the script dictating the appearance of the user-interface displayed. The functionality of the application was coded within a ‘server.R’ file. All three files are required to be run simultaneously (or hosted together online) for operation of the application.

### Data processing and visualisation

**Fig 2:**
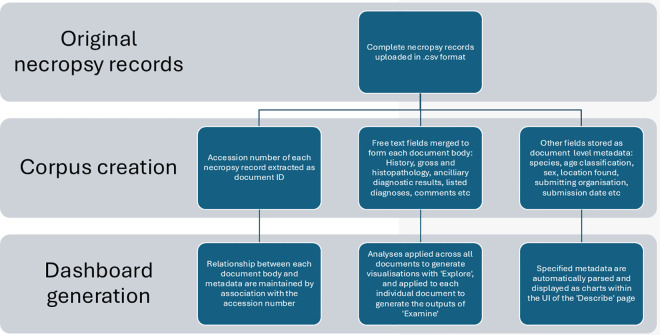
Flow chart outlining the steps of data processing from dataset upload to output generation in an application for extraction and display of information from semi-structured and free-text wildlife necropsy data.

Upon loading, the application opens on the first tab of the *Describe* section titled *Signalment Description.* A sidebar prompts the upload of a.csv file, which needs to be downloaded from the Wildbase Pathology Register prior to starting the workflow. Initial operations within the server.R file combine all free-text fields relating to the findings of each necropsy investigation into a single free-text block for each accession. Each accession is then stored in an object known as a ‘corpus’. Each newly created combined text block now forms the main body of the accession (referred to as the ‘document’) and all other fields are stored as ‘metadata’ to each respective document (termed ‘document-level metadata’) [21] (Fig 2). Species, sex, and age metadata are then rendered into bar charts and tables without pre-processing. Submission date metadata are processed to extract the month and year of each submission. This functionality allows the user to group submissions by month or year creating a visualisation and table which can be toggled between either timespan.

**Fig 3:**
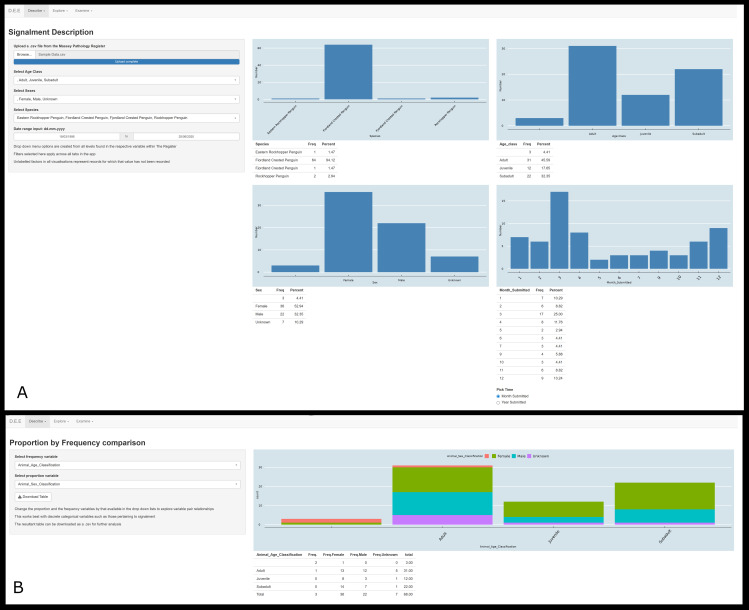
Example display of the *Signalment Description* (top) and *Proportion by Frequency comparison* (bottom) tabs of the *Describe* section of the application for extraction and display of information from semi-structured and free-text wildlife necropsy data. Fig 3a (top) Upon upload of a.csv file downloaded from the Wildbase Pathology Register, a dashboard of selected epidemiologic characteristics of the dataset is displayed alongside a sidebar where this dataset can be filtered by inherent levels within the data. Fig 3b (bottom) Two factor comparison where one variable is represented as proportions (sex classifications) of the variable whose frequency is displayed by the height of the bars (age classifications). The side bar allows selection of any variable stored as metadata to investigate possible relationships. Data presented in these images are derived from randomly compiled accessions and not representative of data within the Wildbase Pathology Register. Both images have been cropped for figure clarity.

The ui.R file organises these visualisations and tables into a two-by-two grid where these data can be overviewed in the *Signalment Description* tab of the *Describe* section (Fig 3a). Discrete levels within each of the metadata variables are simultaneously fed into a dropdown list on the sidebar. For example, the levels of ‘male’, ‘female’, and ’unknown’ may be extracted from the ‘sex’ variable. These lists allow the user to filter the uploaded dataset based on the displayed variables. Filters applied to the uploaded dataset at this stage are maintained throughout the entire functionality of the application. The *Proportion by Frequency comparison* tab of the ‘*Describe’* section allows the user to select any two metadata variables for a two-factor comparison. Here, one variable is represented by colours as proportions of the variable whose frequency is displayed by the height of the bars (Fig 3b).

The aim of the *Describe* section is to overview select demographic and epidemiologic characteristics of the uploaded dataset and allow filtering of these variables dependant of the goals of a user. The example given in Fig 3a shows that most of this random dataset is female by sex classification, and adult by age classification. Further analysis in Fig 3b hints that male juvenile and subadult animals may be underrepresented compared to females of these age classifications. Hypotheses generated from these descriptive visualisations, be they biological mechanisms or sources of bias, can then be tested with more rigorous statistical means or further inquiry.

The *Explore* section also contains two tabs: *Word Correlations* and *Topic Modelling*. Calculation of word correlations identifies terms that frequently co-occur within documents, regardless of their position. These are visualised as a network graph, where line darkness indicates correlation strength. A slider lets users adjust the minimum correlation threshold. Pre-processing removes stop words from each document (e.g., “the”, “and”, “is”), which are excluded throughout the application’s other functions. By inspecting clusters of correlated words, a subject matter expert may be able to infer common themes present throughout the uploaded dataset.

**Fig 4:**
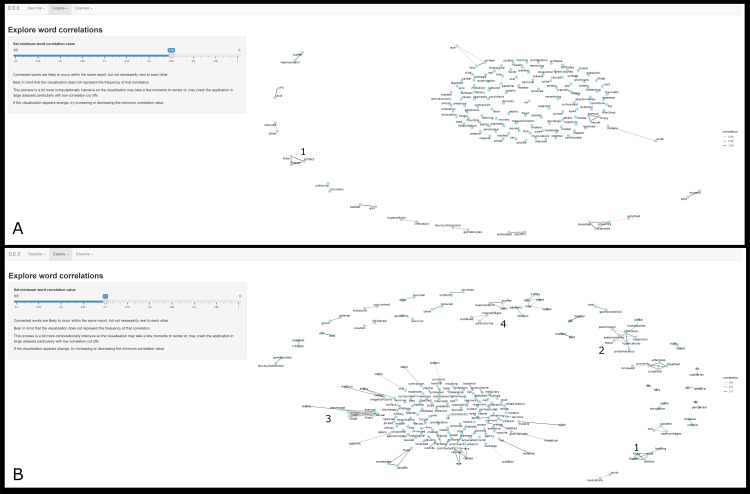
Example displays of the *Word correlations* tab of the *Explore* section of the application for extraction and display of information from free-text wildlife necropsy data. Each point is associated with a word, words that correlate to at least that level specified by the slider in the sidebar are connected by lines. Darker lines represent stronger correlations as detailed by a key on the right. Fig 4a (top) is a sparce visualisation created by a relatively high minimum correlation (0.85) for this dataset. The cluster marked as 1 shows a strong correlation between the words ‘freeze’, ‘thaw’, and ‘artifact’. Fig 4b (bottom) shows a more informative visualisation generated with a lower minimum correlation value (0.7). The cluster marked as 1 is like its equivalent in Fig 4a. Clusters marked 2-4 all contains words which may be interpreted by a subject matter expert as possibly related to plasmodium infection of birds. Data presented in these images are derived from randomly compiled accessions and not representative of data within the Wildbase Pathology Register. The image has been cropped for figure clarity.

The example presented here (Fig 4) shows how adjusting the minimum word correlation value affects the visualisation of this random datafile. Fig 4a shows a relatively high minimum correlation (0.85) and the resultant sparce network graph with few distinct word clusters. However, even in this graph, the cluster marked as ‘1’ (freeze-artifact-thaw) suggests that freezing of specimens prior to submission has occurred in at least some of the records uploaded. In contrast, Fig 4b shows a more informative visualisation created using a lower minimum correlation value (0.7). Here, the cluster marked as ‘1’ is consistent with that in Fig 4a as would be expected, while clusters marked as 2 and 3 both bear some reference to plasmodial organisms. In addition, words like ‘haemosiderin’, ‘kupfer’, and ‘spleen’ seen in the cluster marked as 4 may be interpreted by a subject matter expert as related to common pathological findings in birds infected with plasmodial organisms. Together, these three clusters hint to the possible prevalence of plasmodial infection as relatively common within the uploaded dataset. It should be noted that the precise minimum correlation that will generate a useful visualisation is dependent on both the size of an uploaded dataset and the contents within. As such, this part of the application is meant to be an active, exploratory step.

**Fig 5:**
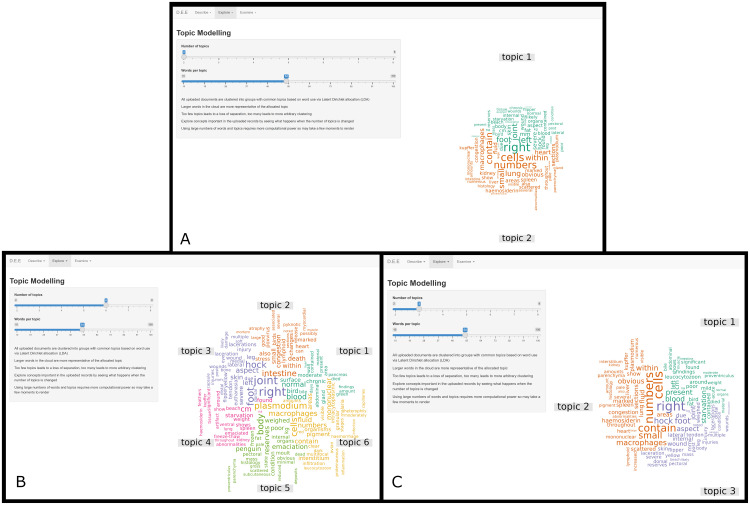
Example displays of the *Topic Modelling* tab of the *Explore* section of the application for extraction and display of information from free-text wildlife necropsy data. Sliders in the sidebar allow the user to manipulate the number of topics and the number of words rendered per topic within the word cloud. 5a shows two topics, 5b shows 6 topics, and 5c shows 3 topics each with equivalent numbers of words per topic. Data presented in these images are derived from randomly compiled accessions and not representative of data within the Wildbase Pathology Register. The image has been cropped for figure clarity.

The specific methodology of topic modelling used in the application is referred to as Latent Dirichlet Allocation (or LDA) [33]. This method of topic modelling is based on three underlying assumptions. Firstly, that individual documents are composed of multiple topics. Secondly, each document exhibits these topics in different proportions. Finally, that these topics are comprised of collections of words which are probabilistically found together. In the process of LDA, the total number of topics within a corpus is the only required input. Once this is set, LDA then calculates the distribution of words over topics [34]. The user-interface then renders this distribution as a ‘word cloud’, where colours differentiate what words are most likely to belong to each topic (Fig 5). By changing the number of topics into which the corpus is divided, the user can actively explore what words often cluster together within and across documents. An appropriate end user (or subject matter expert) may then be able to identify possible contextual relationships between words within a topic which can be investigated further in the *Examine* section of the application.

The example in Fig 5 shows how altering the number of topics can change the appearance of the visualisation and alter a user’s ability to derive insights. In Fig 5a, only two topics are selected. A subject matter expert may interpret the words visible in topic 1 as related to gross pathology, while topic 2 related to histopathology. While it makes intuitive sense that these topics would exist in a repository of necropsy records, it provides no additional information to a user unfamiliar with the dataset. In contrast, Fig 5b contains some topics which hint to trends already discovered within this dataset through the *Word Correlations* tab. Specifically, topic 6 which contains words relating to plasmodial infection. Further information is gleaned from topic 5 which contains the words ‘poor’, ‘body’, and ‘condition’ hinting that emaciation may be a common finding in this dataset. In contrast topic 4 contains relatively non-specific words like ‘bird’, ‘blood’, and ‘surface’, which do not appear to add additional context. Fig 5c is included to demonstrate a potential intermediary step in this admittedly subjective, interrogative process.

The *Examine* section of the application provides the main functionality in terms of identification and quantification of clinicopathologic findings in an uploaded dataset. The user is initially presented with a table collating words identified as having the highest ‘Term Frequency to Inverse Document Frequency’ ratios (TFIDF) in each of the documents. This is calculated by deriving a ratio as to how frequently a term is used in one document to the inverse of how frequently that same term is used across the entire corpus. The underlying principle is that if a term is used very frequently in one document but very sparsely throughout the rest of the corpus, it is likely important to differentiate that one document from the others [21]. By default, the first table collates the top forty TFIDF words from each document in the uploaded corpus. The number of words identified per document can be increased or decreased through use of a slider in the sidebar. Words can be selected from this TFIDF table by left mouse click of the row of interest. Selecting a row triggers the application to then search the corpus to identify all records where that exact word is used in at least one instance, a process referred to as ‘named entity recognition’ [12].

**Fig 6:**
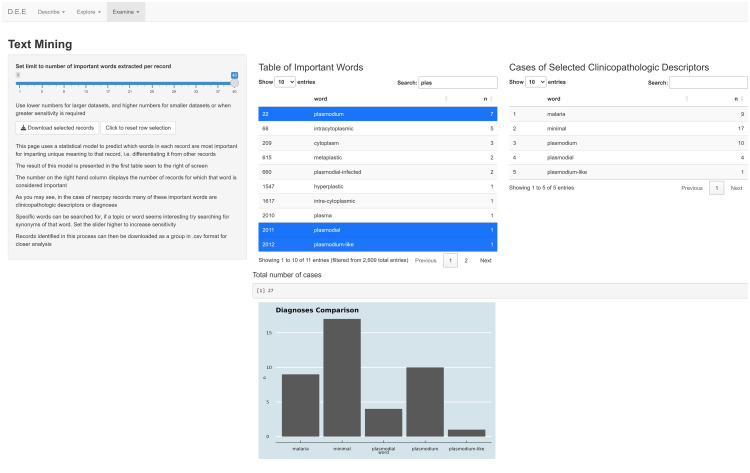
Example display of the only tab, *Text-mining*, in the *Examine* section of the application for extraction and display of information from free-text wildlife necropsy data. The slider in the sidebar allows the user to manipulate the complexity of the ‘Table of Important Words’. Words selected by right click on this table are highlighted in blue. The selected words are then passed through the application’s named entity recognition and negation detection functions. The number of documents where these requirements are met are then transcribed to the table on the right and a histogram of this simultaneously rendered below. Data presented in these images are derived from randomly compiled accessions and not representative of data within the Wildbase Pathology Register. The image has been cropped for figure clarity.

Within the application server, a pipeline exists to exclude circumstances when search terms are used in context with a negation (e.g., ‘no evidence of pneumonia’), through a ‘key-word-in-context’ (KWIC) function. KWIC functions operate much like named entity recognition; however, they also return a ‘window’ of words adjacent to the specified search term, thus providing some ‘context’ from within the resultant records [35]. The pipeline uses a KWIC function to examine each use of the selected search term and only return records where the term is used at least once without a negation in the same ‘phrase’. A ‘phrase’ is defined in the server logic as the first use of punctuation before and after the selected search term within a ten-word window. The selected term and the number of records which fulfil the negation detection criteria are then rendered by the user interface into a second table. Multiple words may be selected from the TFIDF table, after which they will be added to this second table as they are processed by the server. Specific terms of interest can be searched for using a search bar atop the TFIDF table. This search functionality includes partial word matching, allowing a user some ability to identify tense and typographical variants of the same word. This is observed in Fig 6, where ‘plas’ is entered into the search bar, which has allowed the user to select ‘plasmodium’, ‘plasmodial’, and ‘plasmodium-like’. Further, this example shows that variations in terminology can be accounted for by the user also searching for these variants, in this case ‘malaria’ and ‘malarial’. A histogram is simultaneously rendered in the bottom pane of this page providing a visual depiction of this second table. In addition, a text box below the tables reveals the total number of unique records identified in this process. As this is the number of ‘unique’ records, if a record fulfils the negation detection criteria for more than one selected word, it is only counted once. The user is then able to download all these identified records in comma separated value (.csv) format if further investigation is required.

## Discussion

Here we describe the implementation of common text-mining and data wrangling techniques to circumvent many of the barriers that prevent effective and timely data extraction from a passively acquired, free-text based wildlife necropsy database. The application provides wildlife health professionals with a tool to rapidly understand the more common trends in the necropsy database without the need for arduous close reading of reports or deep knowledge of text-mining. Ultimately, the highly variable nature of natural language text means imperfections are expected in some outcomes of the application. However, the secondary analysis by a subject matter expert required to resolve these imperfections, is likely to be a dramatic reduction in workload compared to manually analysing an entire dataset.

*R* is one of the most popular programming languages currently used in bioinformatics [36]. The *Shiny* package allows the creation of web applications within R by bringing its data processing power into what is termed a ‘reactive’ context. Here, ‘reactive’ refers to the package’s ability to change how data is manipulated in accordance with inputs from an end user, without requiring changes in the underlying code. This gives the end user freedom to explore a data set within the bounds of an application built in *Shiny*, while not requiring an understanding of the *R* programming language itself.

As with many published applications developed in *Shiny*, the user experience side of the application presented here was designed around dashboard style displays of information [36]. Derived from the function of automobile dashboards, a data dashboard in this context aims to present information in a simple and interpretable format [37]. The intent is for this consolidated information to then support decision making [38,39]. Effective use of dashboard-style reporting requires an understanding of the potential for creation of implicit biases in the way data is presented [39,40], in addition to those derived from the original dataset itself. Users’ attention may be directed towards certain data while other data is obscured through use of design elements [27], so the purpose and scope of any dashboard must be considered when it is being used as a decision support tool. In this application, aesthetic theming was used judiciously to aid readability. Care was taken to prevent the use of colours and shapes influencing data interpretation [27]. Given dashboards inherently condense information, subtleties in datasets may be lost within the broad trends [27,38]. As such, the application was designed to be used to gain a broad appreciation of an uploaded necropsy dataset, not replace a comprehensive necropsy review.

The application’s stepwise structure mirrors the process commonly used in published necropsy reviews of both captive and free-living wildlife [1,3,4,17]. While each section is designed to be used in sequence, users can navigate freely via a fixed top navigation bar. The *Describe* section was separated into two tabs, one to overview and filter the signalment demographics of the uploaded dataset, then another to explore interdependencies between metadata variables using proportion by frequency plots and tables. These outputs are derived from fundamental concepts in data wrangling (converting data to a useable format) and data exploration (tables and figures) [41].

In traditional manual reviews, it is standard practice to consolidate redundant factor levels (e.g., merging ‘unknown’ and ‘indeterminate’ sex categories) to avoid artificial distinctions.. This step is particularly important if results are to be used as a static display of information (such as in a report to stakeholders). However, exploring data in the raw form not only provides insights into the demographics of the uploaded dataset, but also insights into the use of the Wildbase Pathology Register, providing possible direction on ways to improve data collection and storage. For example, in Fig 3a typographical variation has created redundancy in recording for the ‘Fiordland Crested Penguin’ alongside both ‘Age Classification’ and ‘Sex Classification’ each having three records where this detail is not recorded. Fig 3b then shows that this missing data is distributed as one female missing age classification, one adult missing sex classification, and two records missing both. By observing such idiosyncrasies, data collection efforts can be directed to improve data quality, such as by standardising key inputs. Further, combining factor levels is a process that varies depending on the dataset and the goals of the analysis. Given the diverse applications for which this tool is intended, automating this data-wrangling step could inadvertently introduce a bias in the resultant visualisations. As a result, it was elected to present these data in their original form to preserve data integrity for downstream analyses.

The *Explore* section provides two tools through which the user may gain insights into conceptual patterns occurring between and within the uploaded records: examination of word correlations and unsupervised topic modelling. Examination of clusters and pairs of words in the *Word correlations* tab shows the user which words are most frequently utilised together, providing hints as to commonly occurring concepts within the uploaded records. This relatively simple approach does not consider the frequency or context, with more sophisticated methods available if quantification of the degree of similarity between unknown documents is the primary goal [42]. However, these approaches increase the mathematical burden on what is already a computationally intensive process. As the visualisations generated here are intended to be interpreted by a subject matter expert (as opposed to automatic classification), additional complexity was excluded to make the function more user-friendly.

The second tool within the *Explore* section provides a distinct perspective from which to outline the major contextual relationships between individual, free-text documents of a collection [43]. The broad concept of topic modelling can range from relatively simplistic ‘unsupervised’ processes, to progressively more complex ‘supervised’ approaches where the end user participates in model development [44]. In ‘supervised’ approaches, there is a process of ‘training’ a designed model to classify documents based on previously defined separations. Once the model has been ‘trained’, new documents can then be presented which the model will classify without intervention. As the *Explore* part of the application is designed to review relationships between previously unexamined documents, ‘training’ of a model is not possible; so the unsupervised approach of LDA is used within this application. [45].

Both tabs within the *Explore* section of this application are intended to be an active, interrogative process; whereby the user is encouraged to explore multiple visualisations by adjusting the parameters provided. While these are clearly subjective, qualitative steps of analysis, Figs 4 and 5 demonstrate their potential utility. Careful inspection of the resultant images combined with background knowledge by a subject matter expert can lead to the generation of hypotheses which may be further studied within the application and beyond.

Within the *Examine* section, it is intended that a user applies insights gained from the previous pages and background knowledge to quantify clinicopathologic features of interest. This is achieved through the TFIDF ratio, a method of ‘advanced key-word retrieval’ [12,35] shown to be useful in estimating the degree to which specific words are relevant to documents in various contexts [46]. Within the application, words with a sufficiently high TFIDF ratio are displayed to the user as a table. The user is given exploratory control of this table via the slider in the sidebar. Electing to use a lower number in the slider results in a more concise top TFIDF table and thus an additional method of overviewing common recurring themes in the corpus. The disadvantage of a more concise top TFIDF table is that there is then lowered sensitivity to detecting less frequently used terms or term variants. Given the nature of natural language texts, such terms may be synonyms or typographical variants of words identified as relevant to any research question. In effect, this may reduce the ability of the tool to identify all relevant documents from the corpus [46]. While electing to increase the number of TFIDF words identified solves the issues of using lower numbers, it creates a table that is more complicated to interpret. This more complex table may result in words being included in the table that have no practical value; but rather are identified as having the appropriate TFIDF ratio by mathematical happenstance.

In published processes of advanced key-word retrieval, the words in each document are often ‘stemmed’ prior to any statistical estimations of contextual relevance [12,47,48]. This describes the automatic reduction of inflected words to their ‘stem’ form [12], which need not be an actual word, thus ‘argue’, ‘argued’, and ‘arguing’ may all be stemmed to ‘argu’. The purpose of stemming is to combine the identification of typographical and tense related variants of words so that simply searching for the stemmed word identifies all variants [12], thereby overcoming a limitation of the TFIDF ratio [46]. Stemming is usually carried out automatically, using pre-defined and widely utilised algorithms [12]. In this application, the common method trialled (‘Porter’s algorithm’ within the ‘Snowball C’ package [49]) derived word stems that were often unrecognisable partial words or letter pairs, likely due to idiosyncrasies of clinical and pathologic terminology. This issue is overcome within the application through the search function built into the packages used to render the top TFIDF table. Once the table is rendered, the user has access to a search bar where the entire table can be screened for partial word matches, irrespective of the location within a word that the match occurs, thus replicating the function that stemming may have provided. For example, if the search term is ‘an’, matches presented to the user would include not only words such as ‘anaerobic’ and ‘animal’, but also words such as ‘organism’ and ‘avian’. Further, an appropriate end user can use this functionality to specifically search for synonymous or highly related terms such as ‘malaria’ and ‘plasmodium’ as seen in Fig 6. Such functionality allows the user to then select those word variants of relevance to their particular purpose.

A KWIC function has been used to augment a named entity recognition process, particularly in the detection of contextual negations [50]. Specifically, within natural language clinical reports, suspected findings with no supporting evidence are often explicitly mentioned with a negation via phrases such as ‘no evidence of pneumonia’ [50]. Use of simple ‘named entity recognition’ would identify such cases as a ‘positive’ occurrence of the search term. It is unlikely that cases of such negative reports would be relevant for the purposes of a review of clinical data; so their inclusion may negatively impact the specificity of any search.

Finally, the ability to download all records identified through the processes of the *Examine* section allows manual inspection of these identified records where a more precise case definition may be employed.

## Conclusion

This manuscript presents the development of a dashboard-based application for efficiently interrogating large volumes of free-text wildlife necropsy records. The tool successfully integrates natural language processing and interactive visualisation to support pattern recognition and data exploration. Preliminary use has demonstrated its potential to streamline access to key information across diverse records. While similar methods are well established in other domains, their application to wildlife pathology appears novel. The next phase will involve structured testing by prospective end-users to identify practical constraints and refine usability. While the application itself is restricted to use with the Wildbase Pathology Register, there is considerable potential to adapt the principles used here to other free-text wildlife information repositories. For example, similar tools could be developed for deriving insights from ante-mortem clinical records or even field observation data. This potential leads us to recommend collaboration between wildlife health professionals and data scientists in the construction of necropsy databases and tools for interrogation, to improve the efficiency of utilisation of wildlife necropsy data.

## Supporting information

S1 File Global.R file of the application for extraction and display of information from semi-structured and free-text wildlife necropsy data within the Wildbase Pathology Register. This file contains all the packages used in the development of the application.
(R)

S2 File Server.R file of the application for extraction and display of information from semi-structured and free-text wildlife necropsy data within the Wildbase Pathology Register. This file details the logic behind all the applications functions.
(R)

S3 File Ui.R file of the application for extraction and display of information from semi-structured and free-text wildlife necropsy data within the Wildbase Pathology Register. This file details the user interface of the application.
(R)

S1 Dataset Anonymised dataset of random accessions from the Wildbase Pathology Register. (CSV)
